# miR-18a Inhibits *CDC42* and Plays a Tumour Suppressor Role in Colorectal Cancer Cells

**DOI:** 10.1371/journal.pone.0112288

**Published:** 2014-11-07

**Authors:** Karen J. Humphreys, Ross A. McKinnon, Michael Z. Michael

**Affiliations:** Flinders Centre for Innovation in Cancer, School of Medicine, Flinders University, Flinders Medical Centre, Adelaide, South Australia, Australia; The University of Tennessee Health Science Center, United States of America

## Abstract

The miR-17-92 cluster of microRNAs is elevated in colorectal cancer, and has a causative role in cancer development. Of the six miR-17-92 cluster members, miR-19a and b in particular are key promoters of cancer development and cell proliferation, while preliminary evidence suggests that miR-18a may act in opposition to other cluster members to decrease cell proliferation. It was hypothesised that miR-18a may have a homeostatic function in helping to contain the oncogenic effect of the entire miR-17-92 cluster, and that elevated miR-17-92 cluster activity without a corresponding increase in miR-18a may promote colorectal tumour progression. In colorectal cancer samples and corresponding normal colorectal mucosa, miR-18a displayed lower overall expression than other miR-17-92 cluster members. miR-18a was shown to have an opposing role to other miR-17-92 cluster members, in particular the key oncogenic miRNAs, miR-19a and b. Transfection of HCT116 and LIM1215 colorectal cancer cell lines with miR-18a mimics decreased proliferation, while a miR-18a inhibitor increased proliferation. miR-18a was also responsible for decreasing cell migration, altering cell morphology, inducing G1/S phase cell cycle arrest, increasing apoptosis, and enhancing the action of a pro-apoptotic agent. *CDC42*, a mediator of the PI3K pathway, was identified as a novel miR-18a target. Overexpression of miR-18a reduced *CDC42* expression, and a luciferase assay confirmed that miR-18a directly targets the 3′UTR of *CDC42*. miR-18a mimics had a similar effect on proliferation as a small molecule inhibitor of CDC42. Inhibition of *CDC42* expression is likely to be a key mechanism by which miR-18a impairs cancer cell growth, with a target protector experiment revealing miR-18a influences proliferation via direct inhibition of *CDC42*. Inhibition of *CCND1* by miR-18a may also assist in this growth-suppression effect. The homeostatic function of miR-18a within the miR-17-92 cluster in colorectal cancer cells may be achieved through suppression of *CDC42* and the PI3K pathway.

## Introduction

Disruption of normal miRNA expression levels frequently occurs in colorectal cancer (CRC) development [1]. miRNAs, which are small non-coding RNA sequences, can post-transcriptionally regulate the expression of target genes by binding to complementary target mRNAs. They can cleave complementary mRNAs, or where there is imperfect complementarity, can act through translational inhibition and transcript destabilisation [2]–[4]. While human tumours are often characterised by a general defect in miRNA production and global miRNA down-regulation [5], [6], numerous studies have also shown specific miRNAs to be elevated in CRC [1], [7]. Reduced levels of tumour suppressor miRNAs, or over-expression of oncogenic miRNAs, contribute to tumour progression by altering gene expression and influencing signalling pathways [8], [9]. Indeed, some miRNAs have been shown to be drivers of the oncogenic process, and essential for tumour progression [10]–[13].

One such example of a miRNA with a causative role in cancer development is the miR-17-92 cluster of miRNAs, which has been designated oncomir -1 due to its oncogenic potential [14]. The miR-17-92 cluster, which comprises miR-17, miR-18a, miR-19a, miR-20a, miR-19b, and miR-92a, is commonly elevated in lymphomas and in solid tumours, including colorectal tumours [1], [14]–[17]. The cluster functions during both normal development and oncogenic transformation to promote proliferation and angiogenesis, and inhibit differentiation and apoptosis [11], [12]. miRNAs in the miR-17-92 cluster have also been associated with invasion and metastasis of CRC cells [18], and with poorer survival [19]. The cluster has been shown to coordinate multiple oncogenic pathways, and inhibition of these pathways has therapeutic potential for treating cancers caused by miR-17-92 dysregulation [13].

Of the six miR-17-92 cluster members, miR-19a and b in particular are key promoters of cancer development and cancer cell proliferation [11], [12], [20]. In CRC cells, we have previously shown that of the cluster members, miR-19a and b are responsible for increasing proliferation [20]. Several studies have also shown that miR-19a and b are required and largely sufficient for promoting the oncogenic properties of the cluster in lymphoma models [11], [12]. *In vivo*, miR-19 was required for promoting lymphomagenesis in an Eµ-myc mouse B-cell lymphoma model [11], [12]. miR-19 over-expression led to highly malignant early-onset B lymphomas, whereas disabling miR-19 biogenesis resulted in delayed tumour onset, incomplete penetrance, and extended life span [12]. Following miR-17-92 deletion in lymphoma cells, reintroduction of miR-19 alone restored growth and suppressed apoptosis [11].

In contrast to the pro-proliferative action of miR-19, preliminary evidence suggests that miR-18a may act against the other cluster members to decrease cell proliferation [20]. miR-18a may be less elevated than other members of the miR-17-92 cluster in colorectal tumours [17], and this relative increase in other members of the miR-17-92 cluster could favour increased proliferation. It is known that various post-transcriptional regulatory mechanisms can lead to different levels of individual cluster members, with miR-18a the only member of the cluster to require hnRNPA1 for processing [21]. The tertiary structure of the folded pri-miR-17-92 transcript may also contribute to less efficient processing of miR-18a [22], [23]. We hypothesised that miR-18a may have a homeostatic function in helping to contain the oncogenic effect of the entire miR-17-92 cluster, and that elevated miR-17-92 cluster activity without a corresponding increase in miR-18a may promote colorectal tumour progression. This study aimed to determine the tumour suppressor properties of miR-18a in CRC cell lines.

## Materials and Methods

### Cell culture

HCT116 colorectal carcinoma cells (ATCC, Manassas, VA, USA) were maintained in McCoy's 5A Medium (modified) (Invitrogen, Life Technologies, Carlsbad, CA, USA) containing 10% foetal bovine serum (Bovogen Biologicals, Essendon, VIC, Australia). LIM1215 colorectal carcinoma cells (ECACC, Salisbury, Wiltshire, UK) were maintained in Dulbecco's Modified Eagle's Medium containing 10% foetal bovine serum. Cells were grown at 37°C and 5% CO_2_, maintained at <80% confluence, and were mycoplasma free.

### Human colorectal tissue samples

CRC samples and corresponding normal colorectal mucosa were obtained from the Flinders Tissue Bank (n = 30). These samples were collected by blunt dissection from fresh surgical resections, following ethics approval from the Southern Adelaide Clinical Human Research Ethics Committee and written informed consent from patients. Specific study approval to examine miRNA changes in this colorectal tissue was obtained from the Southern Adelaide Clinical Human Research Ethics Committee (approval number 204.13).

### Transfections

Cell lines were reverse transfected with miRNA mimics, miRNA inhibitors, siRNAs, plasmid contructs, and target protectors using Lipofectamine 2000 (Invitrogen) according to the manufacturer's protocol, in 24 and 96 well plate formats (100 000 and 20 000 cells per well, respectively). For the mimic experiments, miR-18a, miR-19a and b, and negative control (NC) miRNA oligonucleotide duplexes (GenePharma, Shanghai, China) were used at 20 nM each. miR-18a and NC locked nucleic acid (LNA) inhibitors (Exiqon, Vedbaek, Denmark) (IDSs: 18a: 410101-00, NC: 199004-00) were used at 50 nM. Pre-designed Mission siRNAs for *CDC42* (cell division cycle 42) (Sigma-Aldrich, St Louis, MO, USA) (IDs: SASI_Hs01_00222990, SASI_Hs02_00332553) or a NC siRNA (ID: SIC001) were reverse transfected at a total concentration of 20 nM. Co-transfection experiments were performed using 200 ng plasmid DNA (details of constructs below) with 50 nM miR-18a or NC miRNA mimics. Additional co-transfection experiments were performed with 20 nM miR-18a or NC miRNA mimics and with miScript target protectors (Qiagen, Valencia, CA) designed for the miR-18a predicted target gene *CDC42*, or a negative control miScript target protector (ID: MTP0000002). Target protectors were designed for the two potential miR-18a binding sites in the *CDC42* 3′UTR using a Qiagen algorithm, and were reverse transfected at the recommended concentration of 500 nM for each target protector. The target protector context sequence (the region of the 3′UTR flanking the binding site) for the first target site of the *CDC42* 3′UTR was 5′AATGAAGAAAAGTATTGCACCTTTGAAATGCACCAAATGA3′, and for the second target site of the *CDC42* 3′UTR was 5′GTTGAGGTAATCTTTCCCACCTTCCCAAACCTAATTCTTG3′. Additional target protectors were designed for the single potential miR-18a binding site in the *CCND1* 3′UTR (context sequence: 5′TAGATGTGTAACCTCTTCACCTTATTCATGGCTGAAGTCA3′), and experiments were conducted as for *CDC42* above. Cells were cultured for 24–48 h post-transfection.

### Relative quantitation real-time RT-PCR

TRIzol Reagent (Invitrogen) was used to lyse cultured cells and human tissue samples. Total RNA was extracted according to the manufacturer's instructions. RNA was quantified using a Nanodrop-8000 spectrophotometer (Nanodrop Technologies, Wilmington, DE). miRNA expression analysis was conducted by relative quantitation real-time RT-PCR using TaqMan miRNA assays (Applied Biosystems, Life Technologies, Carlsbad, CA, USA). cDNA was synthesized from 20 ng total RNA using miRNA-specific primers according to the TaqMan miRNA Assay protocol, using 3.5 µl master mix and 1.5 µl RT primer in a 7.5 µl final volume. Real-time PCR was carried out according to the TaqMan protocol, using triplicate 10 µl reactions for each biological replicate including 1 µl of reverse transcription product, 0.5 µl miRNA-specific primer and probe assay mix (assay IDs: miR-17: 002308, miR-18a: 002422, miR-19a: 000395, miR-20a: 000580, miR-19b: 000396, miR-92a: 000431), and 1× TaqMan Universal PCR Master Mix No AmpErase UNG (Applied Biosystems). Thermal cycling was performed using a Rotor-Gene Q (Qiagen, Valencia, CA, USA). Results were normalized relative to the endogenous small nuclear RNA, RNU6B (assay ID: 001093). Relative expression levels were calculated from Ct values using Qgene [24].

For mRNA expression analysis, RNA was DNase I treated, and cDNA was synthesized from 1 µg total RNA using M-MLV Reverse Transcriptase, RNase H minus (Promega, Madison, WI, USA) and random hexamer primers in a 25 µl reaction. Real-time PCR was carried out according to the Power SYBR Green protocol (Applied Biosystems) with the following primers for *CDC42*: 5′ACGACCGCTGAGTTATCCAC3′ (forward) and 5′GGCACCCACTTTTCTTTCACG3′ (reverse) and for *CCND1*: 5′GATCAAGTGTGACCCGGACTG3′ (forward) and 5′CCTTGGGGTCCATGTTCTGC3′ (reverse), using triplicate 20 µl reactions including 2 µl of reverse transcription product, 300 nM forward and reverse primers, and 1× Power SYBR Green master mix (Applied Biosystems). Results were normalized relative to endogenous control *ACTB* (β-actin) levels, using the following primers: 5′TTGCCGACAGGATGCAGAAG3′ (forward) and 5′GCCGATCCACACGGAGTACT3′ (reverse), and expression levels calculated using Qgene.

### Western blot analysis

RIPA buffer was used to obtain whole cell protein extracts, which were quantified using an EZQ Protein Quantification kit (Invitrogen). Protein extracts were resolved by SDS–PAGE using pre-cast Mini-PROTEAN TGX Stain-Free Gels (Bio-Rad, Hercules, CA, USA), and electro-blotted onto polyvinylidene difluoride membranes using the Trans-Blot Turbo transfer system and Mini PVDF Transfer Packs (Bio-Rad). Membranes were blocked with 5% bovine serum albumin or skim milk powder in TBS-T prior to overnight incubation with rabbit monoclonal anti-CDC42 (11A11) (1∶1000) (Cell Signaling Technology, Danvers, MA, USA). Rabbit monoclonal anti-GAPDH (glyceraldehyde 3-phosphate dehydrogenase) (D16H11) (1∶1000) (Cell Signalling Technology) was used as a loading control. Secondary horseradish peroxidase-conjugated goat anti-rabbit IgG (Immunopure, Thermo Scientific, Rockford, IL) was used in conjunction with the enhanced chemiluminescence (ECL) system (SuperSignal West Pico, Rockford, IL, USA) to visualize bands using the ChemiDoc MP Imaging system (Bio-Rad). Densitometry results were normalized to GAPDH levels.

### Real time cell growth analysis

Cell proliferation was measured using the xCELLigence RTCA DP instrument (ACEA Biosciences, San Diego, CA, USA). Cells were seeded at 20 000 cells per well of an E-plate and cultured with appropriate treatments. In addition to transfections, treatments also included use of a small molecule inhibitor of CDC42 activity, ML141 (Sigma–Aldrich), at a dose of 20 µM, or a DMSO vehicle control [25], [26]. Growth was measured every 30 min over 48–72 h. The xCELLigence system was also used to measure migration over 24 h using the CIM-plate 16 with cells seeded in serum free media in the top chamber at 30 000 per well, and 10% foetal bovine serum used as an attractant in the bottom chamber. The Incucyte Kinetic Imaging System (Essen BioScience, Ann Arbor, MI, USA) was used as an additional proliferation measure. Cells were seeded at 100 000 cells per well of a 24-well plate, and imaged every hour over 48 h, with 9 images per well. The live-cell phase contrast images were used to calculate confluence using the Incucyte software, and to provide morphology information. In addition to the live-cell imaging, cells were also fixed with formaldehyde at 48 h, and the cytoskeleton was fluorescently stained with Alexa Fluor 488 Phalloidin (Cell Signalling Technology) through the binding of phalloidin to F-actin, with cells imaged using an Olympus BX63 fluorescence microscope with 20× objective (Olympus, Center Valley, PA, USA).

### Caspase apoptosis assays

Apoptosis was measured at 24 h using a Caspase-glo 3/7 assay (Promega) according to manufacturer's instructions, with the luminescent signal proportional to caspase-3/7 activity. The Incucyte also provided an additional real-time measure; the CellPlayer Caspase 3/7 reagent (Essen BioScience) was added to cells at a final concentration of 5 µM, and real-time fluorescent images were taken every hour over 24 h. These images were used to calculate fluorescent cell count, using the IncuCyte Object Counting v2.0 Analysis software. Apoptosis was monitored following transfection with miRNA mimics, with or without additional treatment with the pro-apoptotic agent sodium butyrate (Sigma–Aldrich), at 2.5 mM for 24 h.

### Flow cytometry

Cells were harvested with trypsin 48 h after transfection, washed with PBS, and fixed with 4% formaldehyde with 20 min incubation on ice. Cells were washed with PBS then resuspended in 0.2% triton X in 1% BSA in PBS solution, and incubated at room temperature for 15 min. Cells were washed in 1% saponin wash solution with PBS, then resuspended in PBS/propidium iodide/RNase A and incubated for 30 mins, followed by FACS analysis using a BD FACSCanto II Flow Cytometer (BD Biosciences, San Jose, CA, USA) with PI 680/40 filter.

### miRNA target prediction

Predicted miR-18a target genes were obtained using miRwalk, which collates data from multiple prediction programs (DIANAmT, miRanda, miRDB, miRWalk, PICTAR5, PITA, RNA22, and Targetscan) [27]. Genes common to three or more prediction programs were analysed using Ingenuity Pathway Analysis (Ingenuity Systems, Redwood City, CA) to identify genes involved in proliferation and cell cycle control, and expressed in colorectal cells.

### Plasmid constructs

The *CDC42* 3′UTR (NM_001791.3) was amplified using the following primers: 5′CTCTCCAGAGCCCTTTCTGC3′ (forward) and 5′CAAAGAATTGAGACATGAGAAAGC3′ (reverse), using *PFU* DNA polymerase (Promega) and oligo dT primed cDNA. The 3′UTR was cloned into the pGEM-T Easy vector (Promega), then subcloned into the psiCHECK-2 vector (Promega) using *NotI* restriction sites, and sequenced. psiCHECK-2 contains Renilla luciferase as a primary reporter gene, and Firefly luciferase as an intra-plasmid transfection normalization reporter. The *CDC42* 3′UTR was cloned into the multiple cloning region located downstream of the Renilla translational stop codon, with binding of miRNA to the 3′UTR predicted to result in cleavage and subsequent degradation of the mRNA fusion transcript. Decreased Renilla luciferase activity was used as an indicator of miRNA activity.

### Mutagenesis

Site directed mutagenesis was performed using the QuikChange Lightning Site-Directed Mutagenesis Kit (Agilent Technologies, Santa Clara, CA, USA), according to the manufacturer's instructions, to delete miR-18a seed sequence binding sites in the *CDC42* 3′UTR. Primers for the first target site of the *CDC42* 3′UTR (position 822–844 of NM_001791.3) were: 5′CACTGATAAATGAAGAAAAGTATTGTGAAATGCACCAAATGAATTGAGTT3′ (forward) and 5′AACTCAATTCATTTGGTGCATTTCACAATACTTTTCTTCATTTATCAGTG3′ (reverse). Primers for the second target site of the *CDC42* 3′UTR (position 1295–1317 of NM_001791.3) were: 5′AAAAATGTTGAGGTAATCTTTCCCCCAAACCTAATTCTTGTAGATG3′ (forward) and 5′CATCTACAAGAATTAGGTTTGGGGGAAAGATTACCTCAACATTTTT3′ (reverse). Plasmids were prepared with only the first site mutated, only the second site mutated, or both sites mutated.

### Luciferase assay

Following co-transfection with psiCHECK-2 plasmid constructs and miRNA mimics, luciferase activity was measured after 24 h, using the Dual-luciferase assay kit (Promega), according to manufacturer's instructions. Renilla luciferase activity was normalised to Firefly luciferase activity for the same plasmid.

### Statistical analysis

Data were presented as mean ± standard error of the mean (SEM) of at least three biological replicates, with graphs prepared using GraphPad Prism 6 (GraphPad Software Inc, La Jolla, CA). Statistical analyses were performed using Student's t-test, with a P value <0.05 considered significant.

## Results

### miR-18a has lower overall expression compared with other miR-17-92 cluster members in colorectal cells

**Figure 1:**
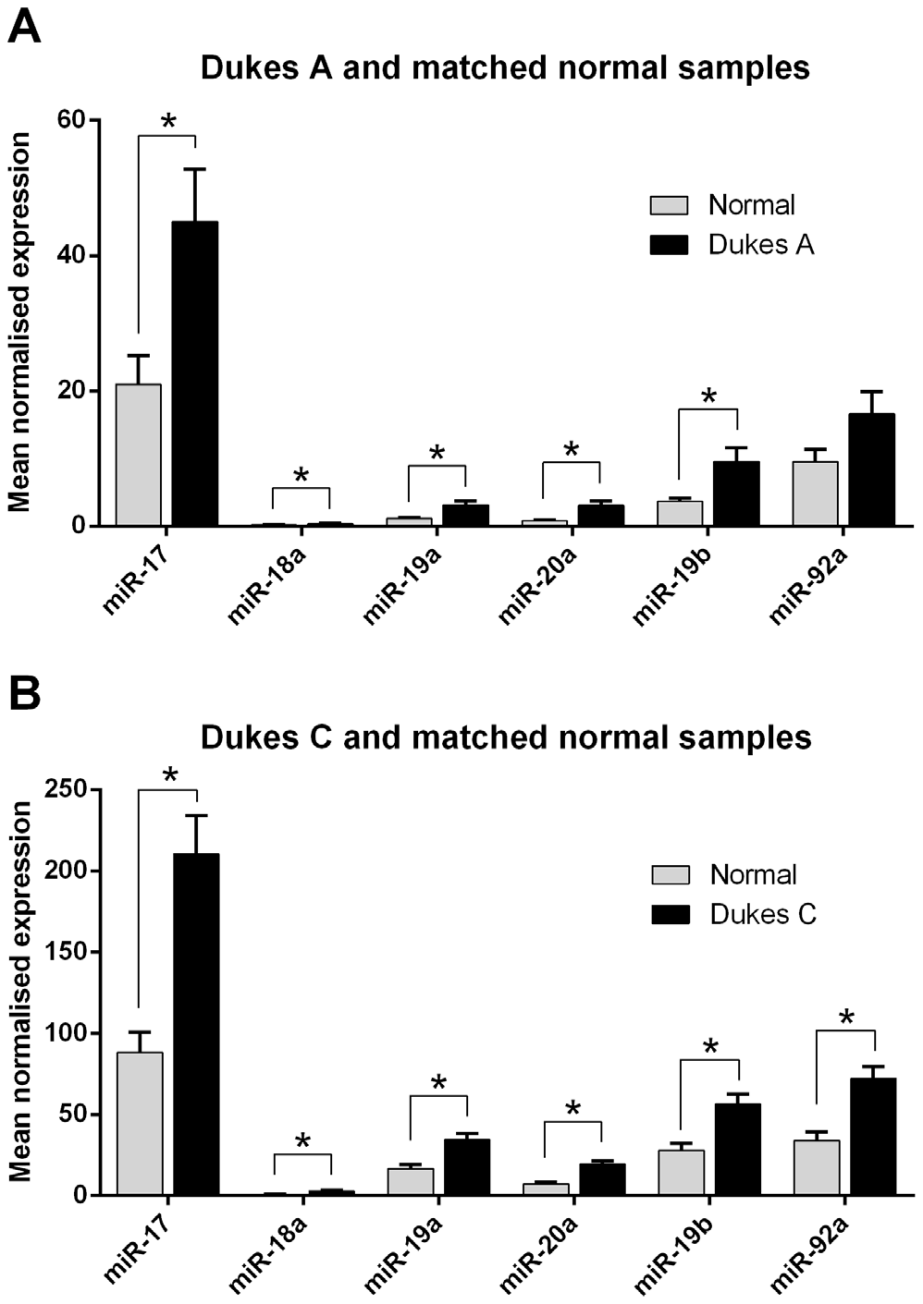
miR-18a has lower overall expression than other miR-17-92 cluster members in CRC and matched normal samples. (A) miR-17-92 cluster miRNA levels in Dukes A and adjacent normal tissue samples (B) miR-17-92 cluster miRNA levels in Dukes C and adjacent normal tissue samples. The mean ± SEM of 30 patients is shown and expression is normalised to RNU6B. * P<0.05.

Human tissue bank samples were used to investigate miR-18a levels in CRC and normal colorectal mucosa, relative to levels of other miR-17-92 cluster miRNAs. miR-17-92 cluster miRNAs levels were quantified in early (Dukes A) and advanced (Dukes C) CRC samples, and corresponding normal colorectal mucosa. miR-17-92 miRNAs were elevated in CRC samples (Dukes A: miR-17 P = 0.01, miR-18a P = 0.01, miR-19a P = 0.005, miR-20a P = 0.002, miR-19b P = 0.009, miR-92a P = 0.07; Dukes C: miR-17 P = 0.0003, miR-18a P = 0.0005, miR-19a P = 0.0009, miR-20a P<0.0001, miR-19b P = 0.001, miR-92a P = 0.0007) compared with the matched normal tissue, and had higher expression in the Dukes C samples (Figure 1A and 1B). miR-18a had lower overall expression compared with other miR-17-92 cluster members, in both the Dukes A and C samples, and in the matched normal tissue (Figure 1A and 1B).

### miR-18a modulates colorectal cancer cell growth and death

**Figure 2:**
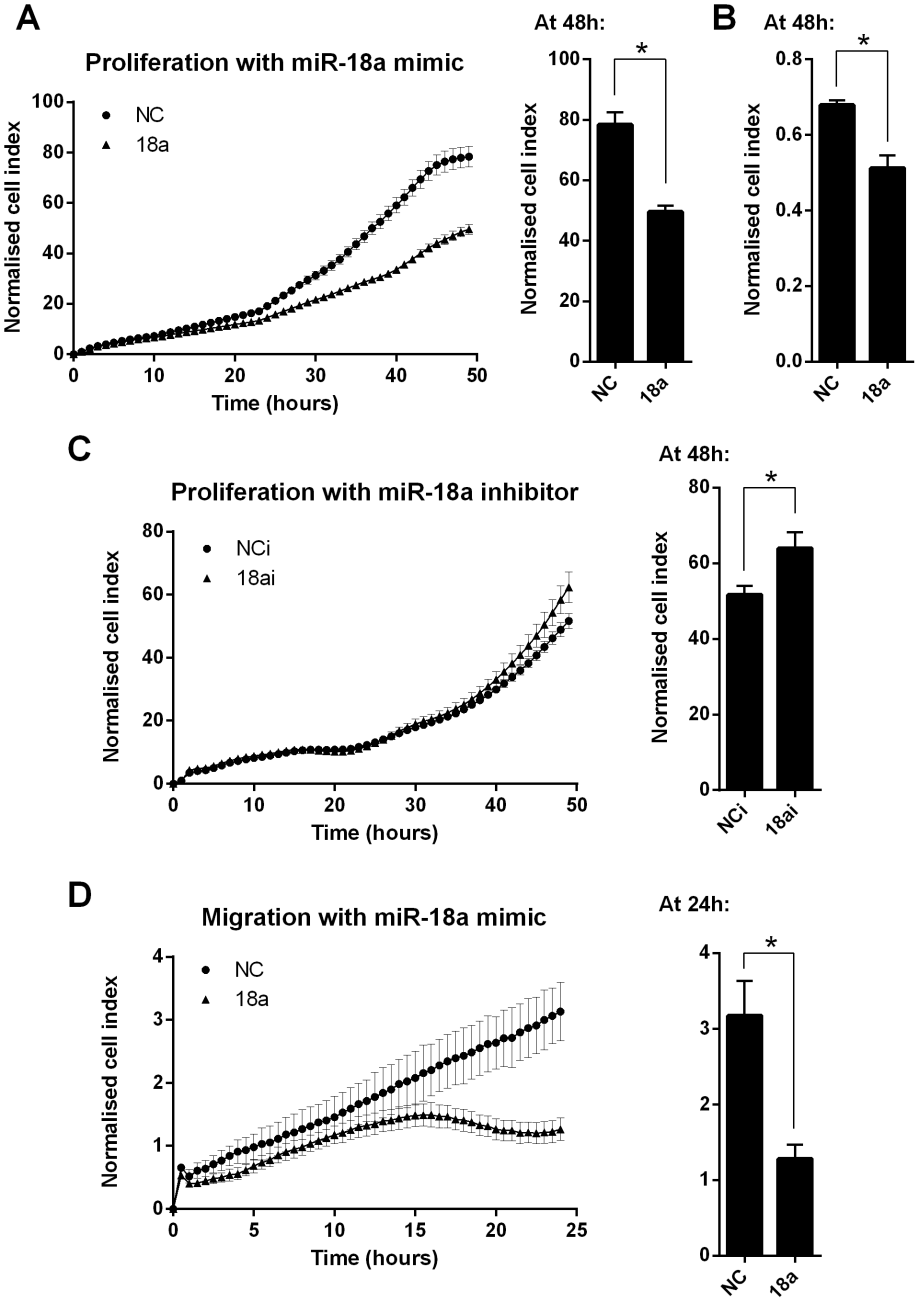
miR-18a reduces cell proliferation and migration in CRC cells. Cell index measurements using the xCELLigence RTCA DP instrument. (A) Proliferation of NC and miR-18a mimic transfected HCT116 cells over 48 h. (B) Proliferation of NC and miR-18a mimic transfected LIM1215 cells over 48 h. (C) Proliferation of NC and miR-18a inhibitor transfected HCT116 cells over 48 h. (D) Migration of NC and miR-18a mimic transfected HCT116 cells over 24 h. The mean ± SEM of 6 cell culture replicates is shown for each. * P<0.05.

Transfection of HCT116 CRC cells with miR-18a mimics led to reduced proliferation over a 48 h time period, compared with the NC mimic transfected cells (P<0.0001) (Figure 2A). A similar anti-proliferative effect for miR-18a was observed in an additional CRC cell line, LIM1215 (P = 0.0009) (Figure 2B). Transfection of HCT116 cells with a miR-18a LNA miRNA inhibitor had the opposing effect, with increased proliferation over a 48 h time period compared with the NC inhibitor transfected cells (P = 0.03) (Figure 2C). HCT116 cells transfected with miR-18a mimics displayed a decreased ability to migrate over a 24 h period, compared with the NC mimic transfected cells (P = 0.004) (Figure 2D).

**Figure 3:**
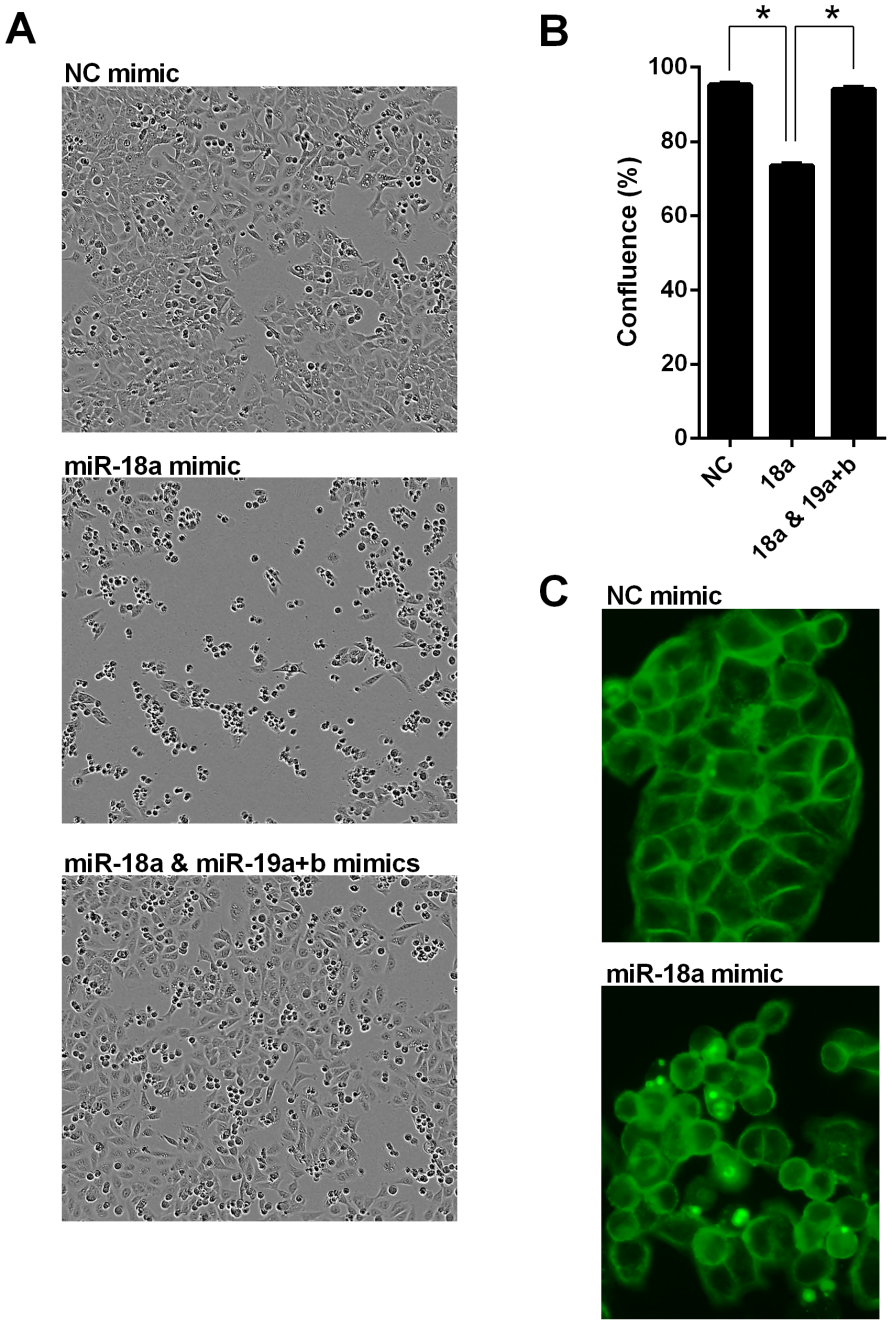
miR-18a alters cell morphology in HCT116 cells. Real-time cell images using the Incucyte instrument. (A) Representative images of NC, miR-18a mimic, and miR-18a, 19a and b mimic transfected cells at 48 h post-transfection. (B) Confluence quantified from real-time images of NC, miR-18a mimic, and miR-18a, 19a and b mimic transfected cells at 48 h. The mean ± SEM of 6 cell culture replicates is shown. * P<0.05. (C) Representative images (20×) of Alexa Fluor 488 Phalloidin fluorescent staining of the actin cytoskeleton of NC and miR-18a mimic transfected cells at 48 h post-transfection.

Transfection with miR-18a mimics also appeared to alter cell morphology. Real-time images taken over 48 h revealed that HCT116 cells transfected with miR-18a mimics were more rounded and less adherent, with a reduced level of confluence (P<0.001) (Figure 3A and 3B). Addition of miR-19a and b mimics largely reversed this effect. Cells transfected with miR-19a and b in addition to miR-18a were closer in appearance to the NC mimic transfected cells, with a more extended morphology, in addition to similar confluence levels over 48 h (P = 0.20); confluence levels significantly increased in these cells compared with the cells transfected with miR-18a mimic alone (P<0.001) (Figure 3A and 3B). Immunofluorescent analysis of the actin component of the cytoskeleton using Alexa Fluor 488 Phalloidin also emphasized the altered cell morphology in the miR-18a mimic transfected HCT116 cells, including a more rounded appearance with reduced intercellular adhesion (Figure 3C).

**Figure 4:**
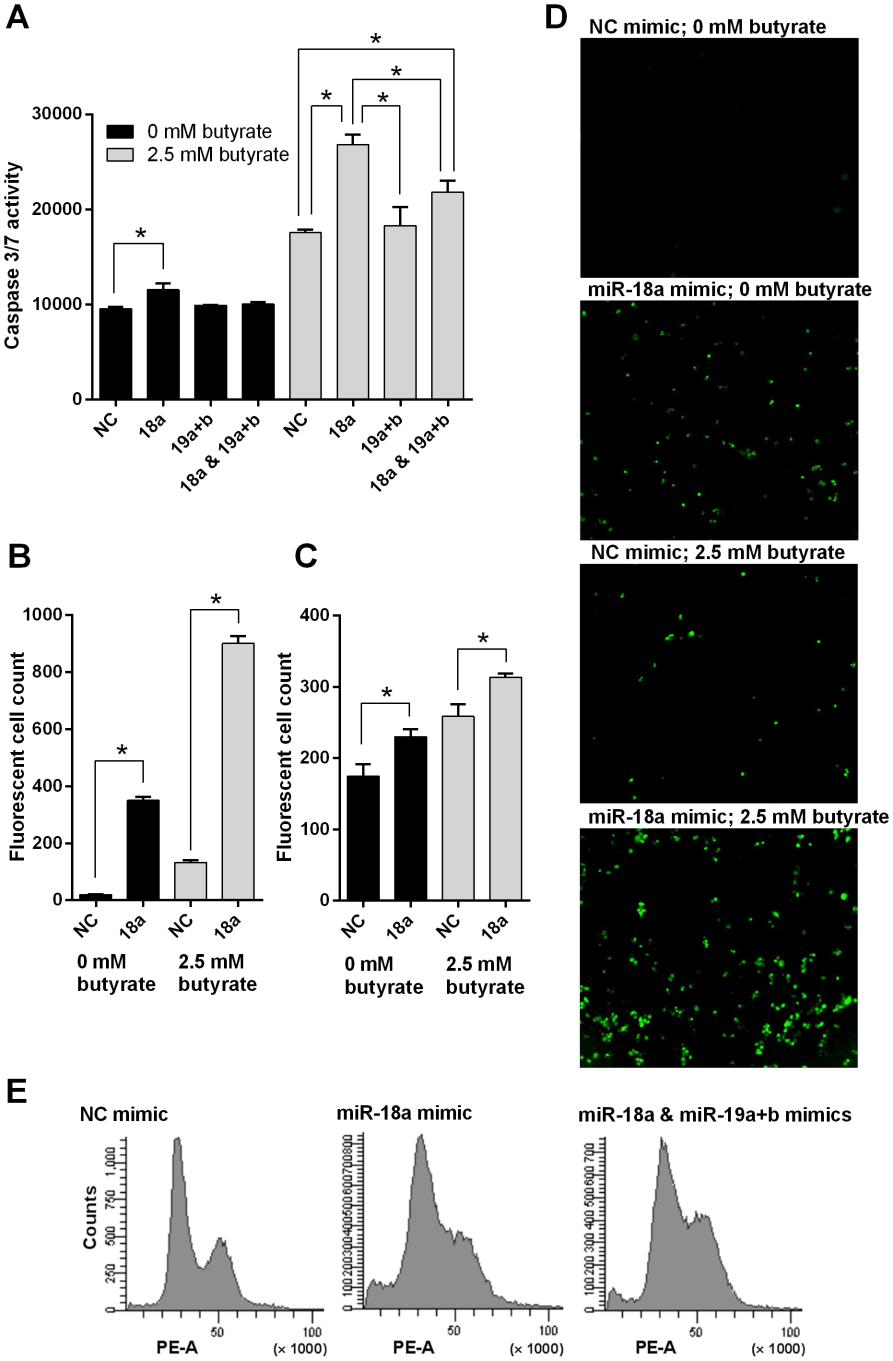
miR-18a induces cell cycle arrest and increases apoptosis in CRC cells. (A) Caspase 3/7 luminescent assay in NC, miR-18a mimic, miR-19a and b mimic, and miR-18a, 19a and b mimic transfected HCT116 cells at 24 h post butyrate addition. The mean ± SEM of 3 cell culture replicates is shown. * P<0.05. (B and D) Quantification and representative real-time fluorescent cell images using the Incucyte instrument showing apoptosis in NC and miR-18a mimic transfected HCT116 cells at 24 h post butyrate addition. The mean ± SEM of 3 cell culture replicates is shown in B. (C) Quantification of real-time fluorescent cell images using the Incucyte instrument showing apoptosis in NC and miR-18a mimic transfected LIM1215 cells at 48 h post butyrate addition. The mean ± SEM of 3 cell culture replicates is shown. (E) Flow cytometry analysis of NC, miR-18a mimic, and miR-18a, 19a and b mimic transfected HCT116 cells at 48 h post-transfection; each graph representative of 3 cell culture replicates.

To measure apoptosis, a caspase 3/7 endpoint assay was performed 24 h after transfection of HCT116 cells. Transfection with miR-18a mimics increased apoptosis compared with NC mimic transfected cells (P = 0.04), whereas co-transfection with miR-19a and b mimics reduced apoptosis compared with miR-18a mimics alone, to a level similar to the NC mimic transfected cells (P = 0.16) (Figure 4A). Transfection with miR-19a and b mimics alone had no effect on apoptosis. Addition of miR-18a mimics also enhanced the action of a known pro-apoptotic agent. HDAC inhibitors, such as butyrate, have previously been shown to induce apoptosis in CRC cells [28]–[30]. Cells transfected with miR-18a mimics in conjunction with 2.5 mM butyrate treatment had higher levels of apoptosis at 24 h compared with the NC mimic transfected cells also treated with butyrate (P = 0.001) (Figure 4A). Again, transfection of miR-19a and b mimics with the miR-18a mimics reduced this effect, compared with the miR-18a mimics alone (P = 0.03), although apoptosis was still higher than with the NC mimics (P = 0.03) (Figure 4A). The effect of miR-18a on apoptosis was further studied in real-time, using the Incucyte system and a fluorescent caspase 3/7 assay to obtain images of cells undergoing apoptosis. When the HCT116 cell images taken 24 h after butyrate treatment were quantified, there were significantly more fluorescent cells with the miR-18a mimic transfection compared with NC mimic transfection, in cells without drug treatment (P<0.001), and in cells treated with 2.5 mM butyrate (P<0.001) (Figures 4B and 4D). A similar effect was observed in the LIM 1215 cells, with differences in fluorescent cell count between the miR-18a mimic and NC mimic transfection reaching significance 48 h after butyrate treatment (P = 0.03 in cells without drug treatment; P = 0.02 in cells treated with 2.5 mM butyrate) (Figure 4C).

Flow cytometry was performed 48 h after transfection of HCT116 cells, and also showed an accumulation of cells undergoing cell death or apoptosis in the miR-18a mimic transfected cells (Figure 4E). The miR-18a mimics were shown to induce G1/S phase cell cycle arrest. Addition of miR-19a and b mimics resulted in decreased cell death and cell cycle arrest than with miR-18a mimic alone (Figure 4E).

### Expression of the cell cycle control gene CDC42 is decreased by miR-18a and increased by miR-19a and b

Given the findings that miR-18a reduces proliferation and migration, alters cell morphology, increases apoptosis, and induces cell cycle arrest, predicted miR-18a targets were identified that are involved in these cellular processes. Among miR-18a predicted targets was cell division cycle 42 (*CDC42*), which was predicted by multiple target prediction programs. CDC42 is a member of the Rho family of GTPases, a subfamily of the Ras superfamily of small GTPases [31]. Upon activation, CDC42 is able to bind a variety of effector proteins and initiate numerous downstream signalling pathways, including those involved in cytoskeletal remodelling, cell cycle progression, cell proliferation, survival and migration [32]–[37]. There are several transcript variants of *CDC42*, which encode a canonical isoform present in most tissues (isoform 1), and a brain-specific isoform (isoform 2). The 3′UTR of the transcripts for the canonical isoform contains two predicted miR-18a binding sites, while the alternate 3′UTR of the transcript for the brain isoform contains two different predicted miR-18a binding sites. For the purpose of this study in colorectal cells, *CDC42* isoform 1 was investigated.

**Figure 5:**
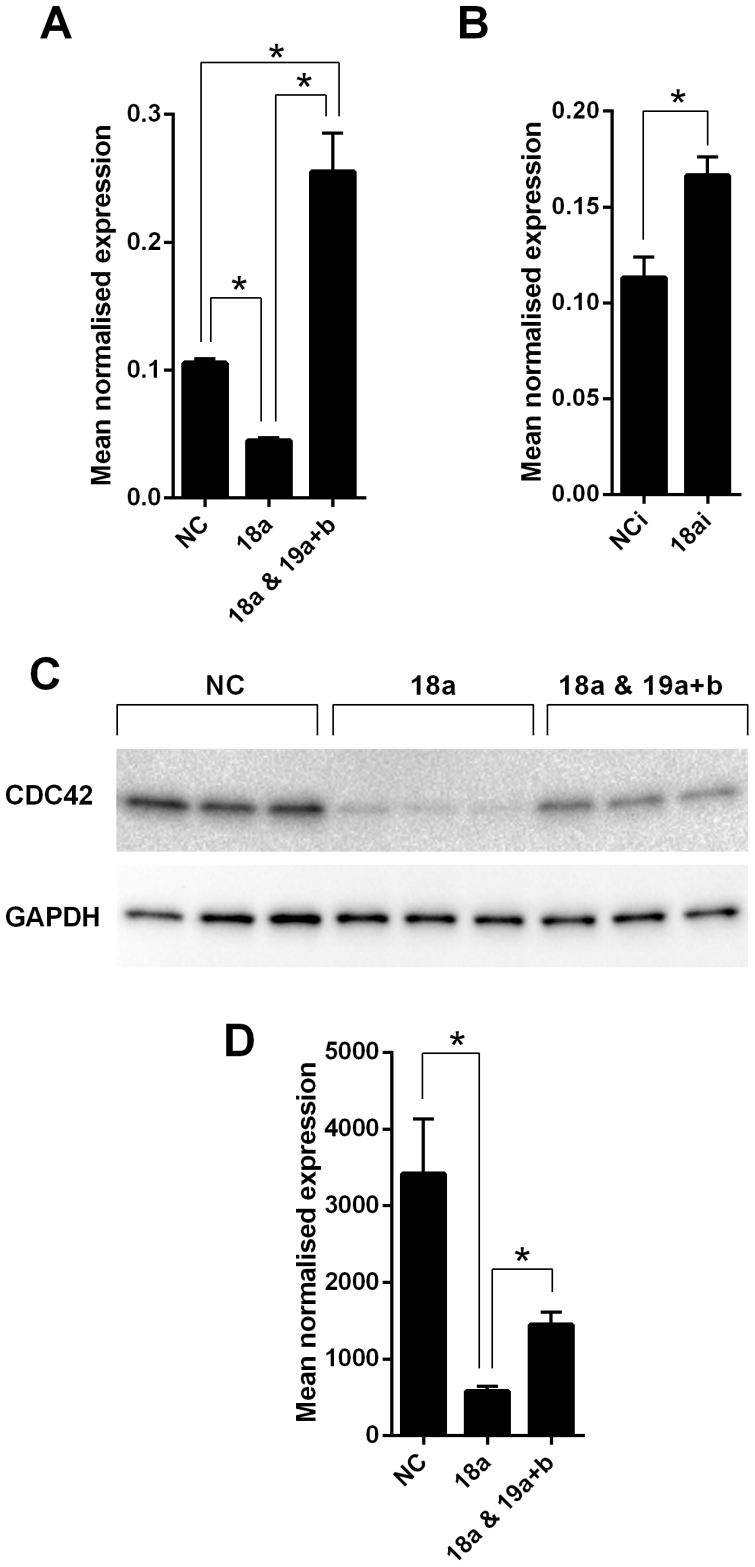
*CDC42* expression is reduced by miR-18a but increased by miR-19a and b in HCT116 cells. (A) *CDC42* mRNA levels in NC, miR-18a mimic, and miR-18a, 19a and b mimic transfected cells at 48 h post-transfection (B) *CDC42* mRNA levels in NC and miR-18a inhibitor transfected cells at 48 h post-transfection. The mean ± SEM of 6 cell culture replicates is shown for each and expression is normalised to ACTB. * P<0.05. (C and D) Western blot of CDC42 protein levels in NC, miR-18a mimic, and miR-18a, 19a and b mimic transfected cells at 48 h post-transfection. Densitometry graph shows the mean ± SEM of 3 cell culture replicates for each and expression is normalised to GAPDH * P<0.05.

Transfection of HCT116 CRC cells with miR-18a mimics reduced transcript levels of *CDC42* compared with the NC mimic transfected cells at 48 h, when detected by real-time RT-PCR (P<0.0001) (Figure 5A). Surprisingly, co-transfection of miR-19a and b mimics along with the miR-18a mimics produced the opposite effect, with increased *CDC42* mRNA levels that were significantly higher than both the NC mimic cells (P = 0.0006) and the miR-18a mimic transfected cells (P<0.0001) (Figure 5A). Transfection with a miR-18a inhibitor increased transcript levels of *CDC42* compared with the NC mimic transfected cells at 48 h, when detected by real-time RT-PCR (P<0.005) (Figure 5B).

Western blot analysis showed CDC42 protein levels were also significantly reduced in the miR-18a mimic transfected cells compared with the NC mimic transfected cells, at 48 h (P = 0.02) (Figure 5C and 5D). Co-transfection of miR-19a and b mimics with the miR-18a mimics increased protein levels compared with miR-18a mimics alone (P = 0.008), with levels closer to that of NC mimic transfected cells (P>0.05) (Figure 5C and 5D).

### miR-18a directly targets the 3′UTR of CDC42

**Figure 6:**
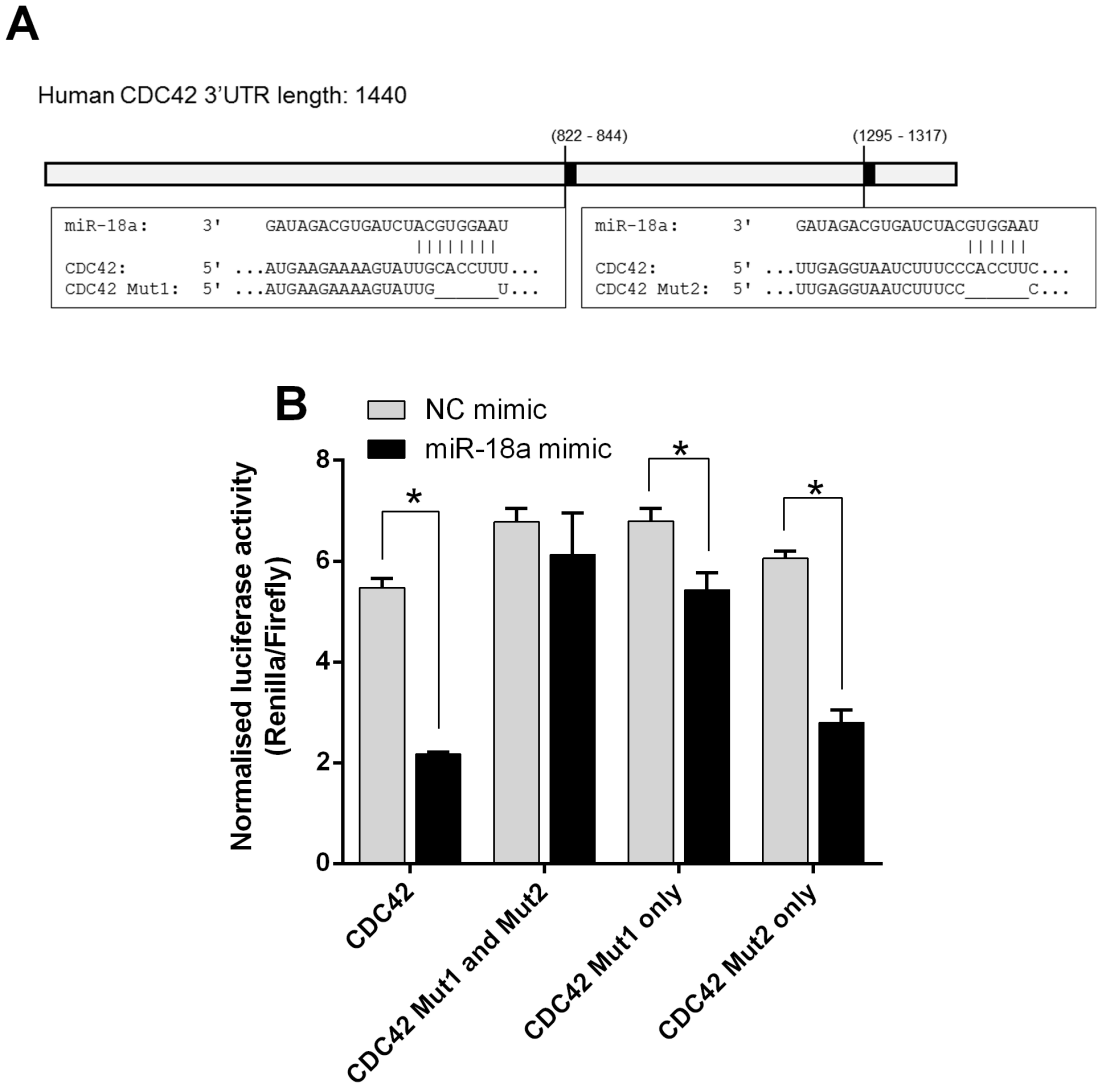
The *CDC42* 3′UTR is directly targeted by miR-18a. (A) Predicted miR-18a binding sites in the *CDC42* 3′UTR (NM_001791.3). The mutated *CDC42* 3′UTR sequence for each binding site is also shown. (B) Normalised Renilla luciferase activity in HCT116 cells with intact or mutated *CDC42* 3′UTR psiCHECK-2 plasmid construct (both miR-18a binding sites mutated, first site mutated, or second site mutated), and with NC or miR-18a mimics, at 24 h post-transfection. The mean ± SEM of 4 cell culture replicates is shown. * P<0.05.

A luciferase assay system was used to determine that miR-18a directly targets the *CDC42* 3′UTR. Dual-luciferase reporter plasmids were constructed with an intact *CDC42* 3′UTR, or with *CDC42* 3′UTRs mutated at the first, second, or both predicted miR-18a binding sites (Figure 6A). HCT116 cells were transfected with each plasmid, in conjunction with miR-18a or NC mimics, and Firefly and Renilla luciferase activity were measured at 24 h. A decrease in normalised Renilla luciferase activity was used as an indicator of miR-18a binding and repression. The cells with intact *CDC42* 3′UTR plasmid showed significantly reduced luciferase activity with the co-transfected miR-18a mimic compared with the NC mimic (P<0.0001) (Figure 6B). Mutation of only the first target site partially restored luciferase activity in the presence of miR-18a mimic compared with NC mimic (P = 0.02). Mutation of only the second target site was largely unable to restore luciferase activity, which was still significantly reduced in the presence of the miR-18a mimic compared with the NC mimic (P<0.0001). With both target sites mutated, luciferase activity was similar with the miR-18a and NC mimics (P = 0.48) (Figure 6B). The first predicted target site appears more responsible than the second target site for miR-18a binding and repression; however, mutation of both sites was required to completely prevent the action of miR-18a.

### miR-18a influences cell growth by inhibiting CDC42

**Figure 7:**
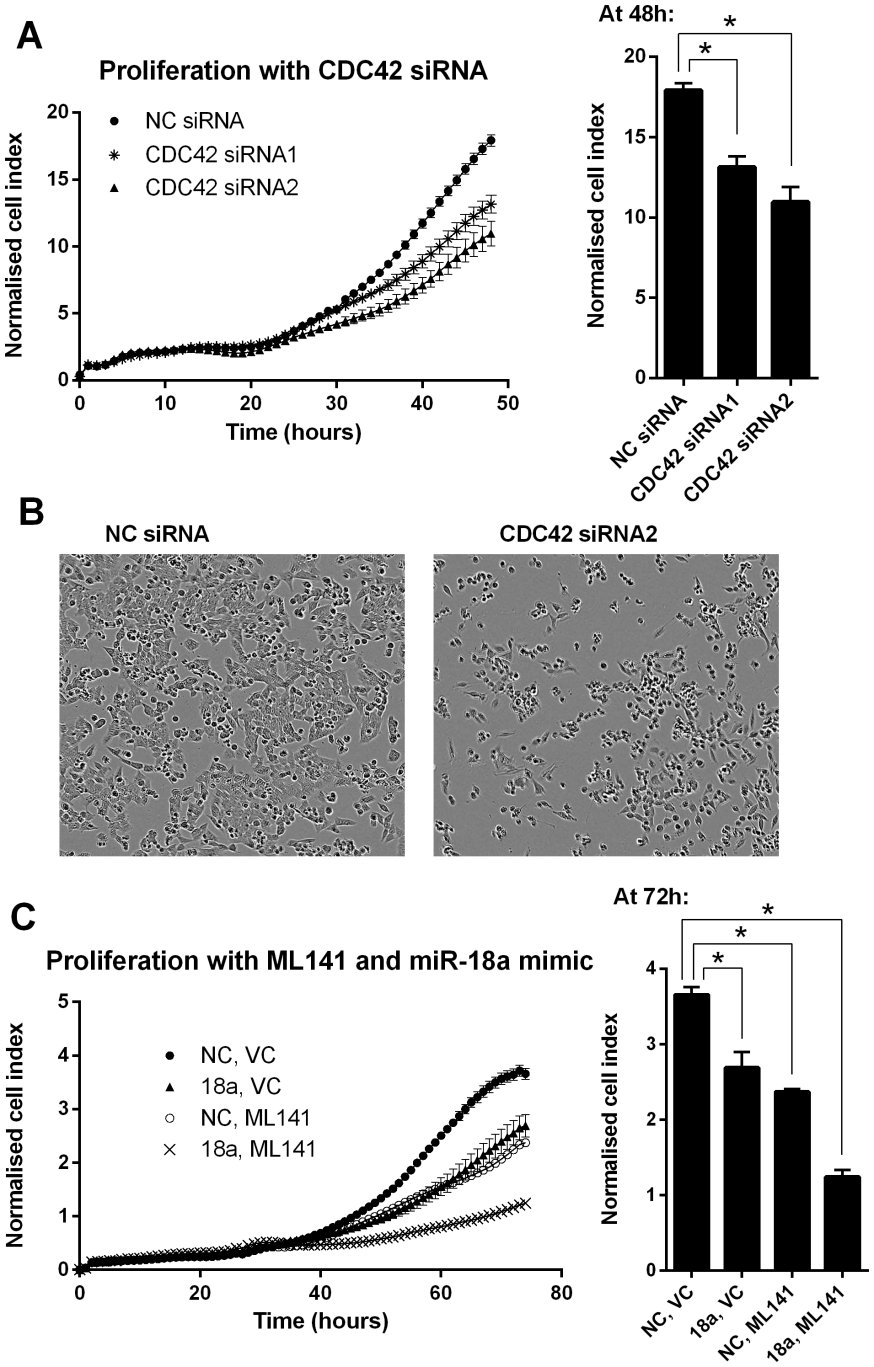
*CDC42* siRNAs and CDC42 protein inhibitor, ML141, have similar effects on cell proliferation and morphology as miR-18a in HCT116 cells. (A) Cell index measurements using the xCELLigence RTCA DP instrument showing proliferation of cells transfected with NC siRNA or two different *CDC42* siRNAs over 48 h. The mean ± SEM of 6 cell culture replicates is shown for each. * P<0.05. (B) Representative real-time cell images using the Incucyte instrument showing NC siRNA and *CDC42* siRNA transfected cells at 48 h post-transfection. (C) Cell index measurements using the xCELLigence RTCA DP instrument showing proliferation of cells transfected with NC or miR-18a mimics over 72 h, with the addition of ML141 or vehicle control (VC) at 24 h. The mean ± SEM of 4 cell culture replicates is shown for each. * P<0.05.

Transfection of HCT116 cells with two different *CDC42* siRNAs produced similar cellular effects as for the miR-18a mimic transfection, including decreased proliferation compared with a NC siRNA at 48 h (P = 0.0001 for *CDC42* siRNA1; P<0.0001 for *CDC42* siRNA2) (Figure 7A), and altered cell morphology with more rounded cells observed (Figure 7B).

A recently identified small molecule that inhibits CDC42 protein activity by preventing GTP binding has been shown to be selective and potent in biochemical and cellular assays [26]. HCT116 cells were treated with this inhibitor, at a dosage previously determined to selectively inhibit CDC42 activity by ∼50% [25]. This small molecule inhibitor of CDC42 had a similar effect on proliferation as the miR-18a mimics at 72 h (P = 0.006 for miR-18a mimic and P<0.001 for ML141 compared with the NC mimic vehicle only control), and together these two treatments had a synergistic effect and further decreased proliferation (P<0.001 compared with the NC mimic vehicle only control) (Figure 7C).

**Figure 8:**
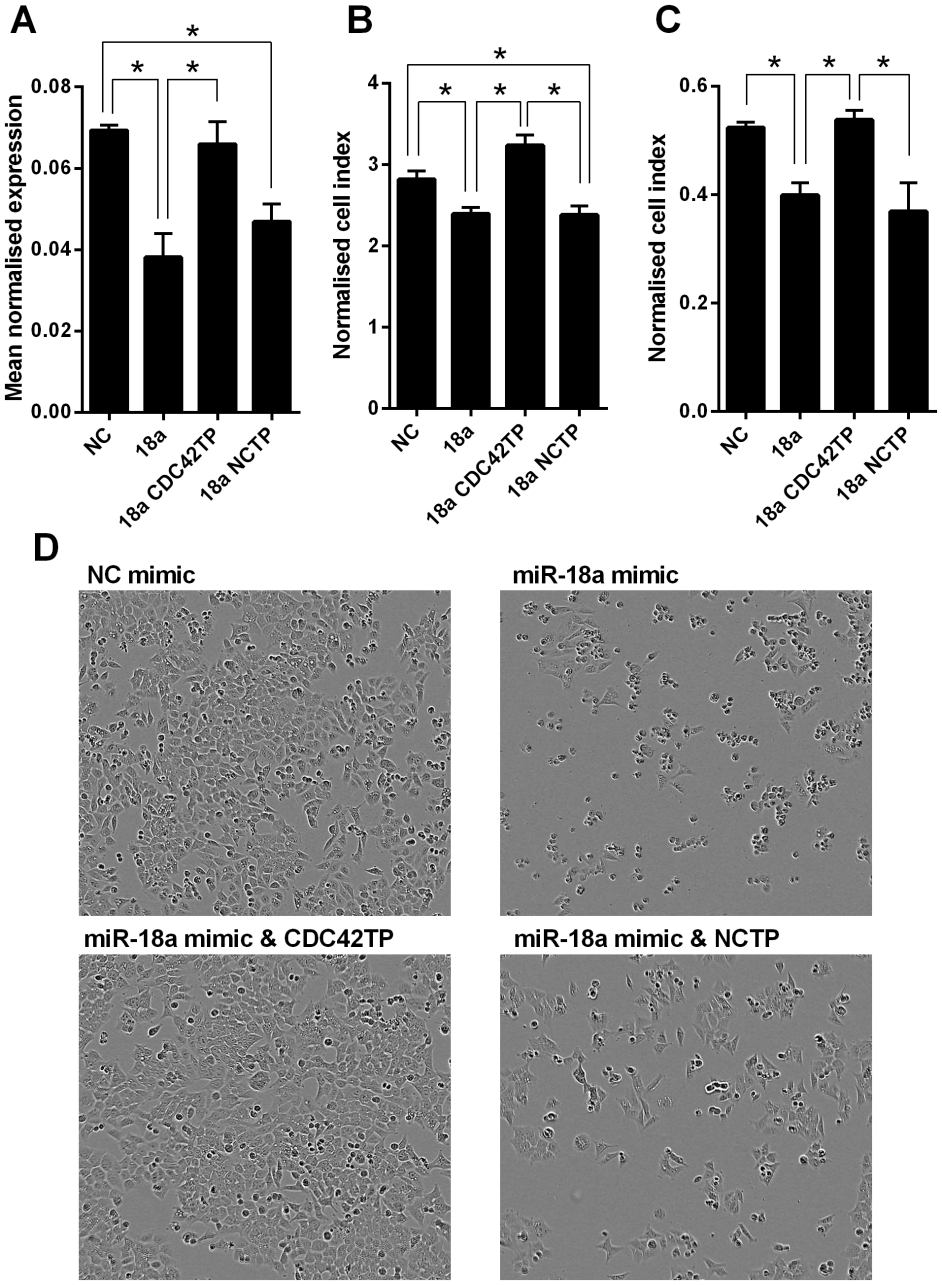
Inhibition of *CDC42* is a mechanism by which miR-18a reduces cell growth in CRC cells. (A) *CDC42* mRNA levels in NC mimic, miR-18a mimic, miR-18a mimic plus *CDC42* target protectors (CDC42TP), and miR-18a mimic plus NC target protector (NCTP) transfected HCT116 cells at 48 h post-transfection. The mean ± SEM of 3 cell culture replicates is shown for each and expression is normalised to ACTB. * P<0.05. (B and C) Cell index measurements using the xCELLigence RTCA DP instrument showing proliferation of HCT116 (B) and LIM1215 (C) cells transfected with NC mimic, miR-18a mimic, miR-18a mimic plus *CDC42* target protectors (CDC42TP), and miR-18a mimic plus NC target protector (NCTP) at 48 h post-transfection. The mean ± SEM of 3 cell culture replicates is shown. * P<0.05. (D) Representative real-time cell images using the Incucyte instrument of NC mimic, miR-18a mimic, miR-18a mimic plus *CDC42* target protectors (CDC42TP), and miR-18a mimic plus NC target protector (NCTP) transfected HCT116 cells at 48 h post-transfection.

Target protectors were used to confirm that the direct targeting of the *CDC42* 3′UTR by miR-18a is a mechanism for the reduced cell growth observed with high miR-18a levels. These single-stranded, modified RNAs specifically interfere with the interaction of a miRNA with a single endogenous mRNA target site, while leaving the regulation of other targets of the same miRNA unaffected. Target protectors were designed for the two miR-18a target sites in the *CDC42* 3′UTR. Transfection of HCT116 cells with miR-18a mimics decreased *CDC42* mRNA levels compared with a NC mimic at 48 h (P = 0.007); however, use of *CDC42* target protectors in addition to the miR-18a mimics prevented miR-18a mediated reduction of *CDC42* mRNA levels. mRNA levels were higher than with the miR-18a mimic alone (P = 0.03), and were similar to levels in the NC mimic transfected cells (P>0.05) (Figure 8A). In HCT116 cells, the miR-18a mimic decreased cell growth compared with a NC mimic at 48 h (P = 0.0003), whereas use of the *CDC42* target protectors in addition to miR-18a mimic restored growth to at least that of the NC mimic at 48 h (P>0.05) (Figure 8B and 8D). Growth was significantly decreased with the miR-18a mimic alone compared with the miR-18a mimic plus *CDC42* target protector co-transfection (P<0.0001); miR-18a thus appeared to decrease cell growth by directly inhibiting *CDC42*, with the specific target protectors blocking this inhibition and subsequent growth effect. Conversely, the NC target protector was unable to protect *CDC42* from miR-18a binding and regulation (Figure 8A), with the miR-18a mimic plus NC target protector co-transfection reducing growth compared with the NC mimic (P = 0.006), and compared with the miR-18a plus *CDC42* target protector co-transfection (P = 0.001), to a similar extent as with the miR-18a mimic alone (Figure 8B and 8D). A similar effect was observed in the LIM1215 cells, with miR-18a unable to produce its growth inhibitory effect in the presence of *CDC42* target protectors (Figure 8C).

### miR-18a also influences cell growth by inhibiting CCND1

**Figure 9:**
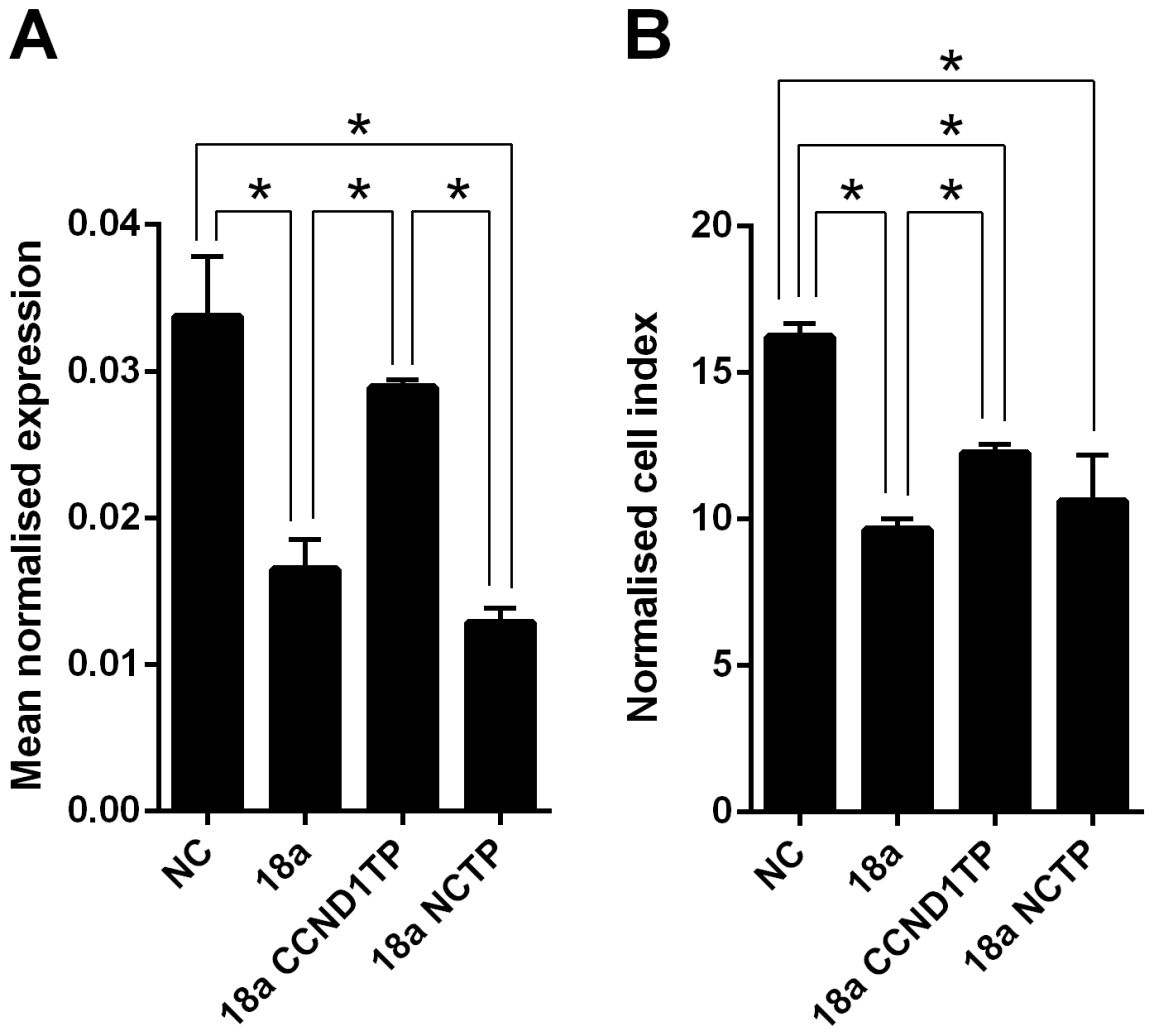
Inhibition of *CCND1* is an additional mechanism by which miR-18a reduces cell growth in CRC cells. (A) *CCND1* mRNA levels in NC mimic, miR-18a mimic, miR-18a mimic plus *CCND1* target protectors (CCND1TP), and miR-18a mimic plus NC target protector (NCTP) transfected HCT116 cells at 48 h post-transfection. The mean ± SEM of 3 cell culture replicates is shown for each and expression is normalised to ACTB. * P<0.05. (B) Cell index measurements using the xCELLigence RTCA DP instrument showing proliferation of HCT116 cells transfected with NC mimic, miR-18a mimic, miR-18a mimic plus *CCND1* target protectors (CCND1TP), and miR-18a mimic plus NC target protector (NCTP) at 48 h post-transfection. The mean ± SEM of 3 cell culture replicates is shown. * P<0.05.

As miRNAs may regulate hundreds of target genes, it is likely that miR-18a is achieving its growth-suppression action through other targets in addition to *CDC42*. Other important proliferation and cell cycle regulation genes were examined, to identify additional miR-18a predicted targets. Of these, the cell-cycle progression gene, *CCND1*, was predicted by multiple target prediction programs to have a miR-18a target site. Target protectors were designed for the miR-18a target site in the *CCND1* 3′UTR. Transfection of HCT116 cells with miR-18a mimics decreased *CCND1* mRNA levels compared with a NC mimic at 48 h (P = 0.02), while use of *CCND1* target protectors in addition to the miR-18a mimics prevented miR-18a mediated reduction of *CCND1* mRNA levels, with levels similar to the NC mimic (P>0.05) (Figure 9A). In the HCT116 cells, growth was significantly decreased with the miR-18a mimic alone compared with the miR-18a mimic plus *CCND1* target protector co-transfection (P = 0.004) (Figure 9B). Unlike the results for the *CDC42* target protector experiments, however, the *CCND1* target protector was unable to completely reverse the growth-suppression effect of miR-18a, with the 18a mimic plus *CCND1* target protector co-transfection still showing lower growth than the NC mimic (P = 0.002) (Figure 9B).

## Discussion

miRNAs are key regulators of cancer progression, with this study indicating that miR-18a has a tumour suppressor role in CRC. miR-18a mimics were shown to decrease proliferation in CRC cells, while a miR-18a inhibitor increased proliferation. miR-18a was also found to be responsible for decreasing cell migration, altering cell morphology, inducing G1/S phase cell cycle arrest, and increasing apoptosis, both alone and in the presence of a pro-apoptotic agent.

miR-18a was found to directly target the 3′UTR of *CDC42*, and to reduce *CDC42* expression. This regulation could explain some of the observed down-stream cellular effects of miR-18a. CDC42 is an important regulator of cytoskeletal remodelling (including filopodia formation and microtubule stabilisation), cell cycle progression (promoting G1 to S phase transition), cell proliferation, survival and migration [32]–[37]. Increased *CDC42* expression has been observed in multiple human cancers, including CRC [38], and elevated CDC42 may also contribute to cancer therapy resistance [39]. Recent evidence points to CDC42 activation as a crucial step in malignant progression in colorectal cells [40]. In our study, the *CDC42* RNA interference phenocopied the miR-18a mimic transfections, with decreased proliferation and altered morphology evident. Thus, inhibition of *CDC42* expression is likely to be a key mechanism by which miR-18a impairs cancer cell growth. Preventing miR-18a binding with target protectors confirmed that direct targeting of the *CDC42* 3′UTR by miR-18a is a mechanism for the reduced cell growth observed with high miR-18a levels; when the *CDC42* mRNA was shielded from miR-18a suppression this growth reduction was no longer possible. miR-18a also reduced mRNA levels of *CCND1*; CCND1 plays an important role in promoting cell cycle progression, and is frequently overexpressed in cancer, including CRC [41]. When the *CCND1* mRNA was shielded from miR-18a suppression using a target protector, the effect of miR-18a-restrained proliferation was partially reduced; however, unlike the results for the *CDC42* target protectors, the *CCND1* target protector was unable to completely reverse growth suppression. It appears likely that miR-18a achieves growth suppression through the cumulative inhibition of multiple target genes, with some targets (particularly *CDC42*) playing greater roles than others (such as *CCND1*).

There is emerging evidence that components of the miR-17-92 cluster antagonize each other, in order to maintain balance between oncogenic and tumour suppressor pathways, in various cell contexts. While the miR-17-92 cluster as a whole acts as an oncogene, Olive et al (2013) found that deleting miR-92a increased the tendency of the miR-17-92 gene to promote Burkitt's lymphoma, with miR-92a and the oncogenic miR-19a and b opposing each other in lymphoma development [42]. Colorectal cancer progression may be associated with elevated miR-17-92 transcription without a corresponding increase in mature miR-18a. In this study, while all miR-17-92 miRNAs, including miR-18a, were elevated in CRC samples, miR-18a levels remained low relative to the other cluster miRNAs. Elevation of miR-18a activity through the use of mimics led to deceased proliferation, cell morphology changes, apoptosis, and cell cycle arrest; however, addition of miR-19a and b mimics reversed these effects, and the cells had a more aggressive phenotype similar to the NC transfected cells. miR-18a was shown to directly target the *CDC42* 3′UTR to decrease mRNA levels, and thereby decrease protein, while miR-19a and b increased *CDC42*. There are several potential mechanisms by which miR-19a and b could increase *CDC42* mRNA levels. It is possible that miR-19a and b could bind to the *CDC42* promoter region and increase transcription. Studies in other miRNA and gene combinations establish a precedent for this; for example, miR-373 has been shown to induce expression of genes with complementary promoter sequences [43]. However, target prediction using miRwalk did not reveal any obvious target sites for miR-19a and b in the *CDC42* promoter region. Alternatively, miR-19a and b could be supressing an inhibitor of *CDC42* transcription. Regulation of *CDC42* transcription remains relatively unstudied, while activation of CDC42 protein is better characterised [44].

**Figure 10:**
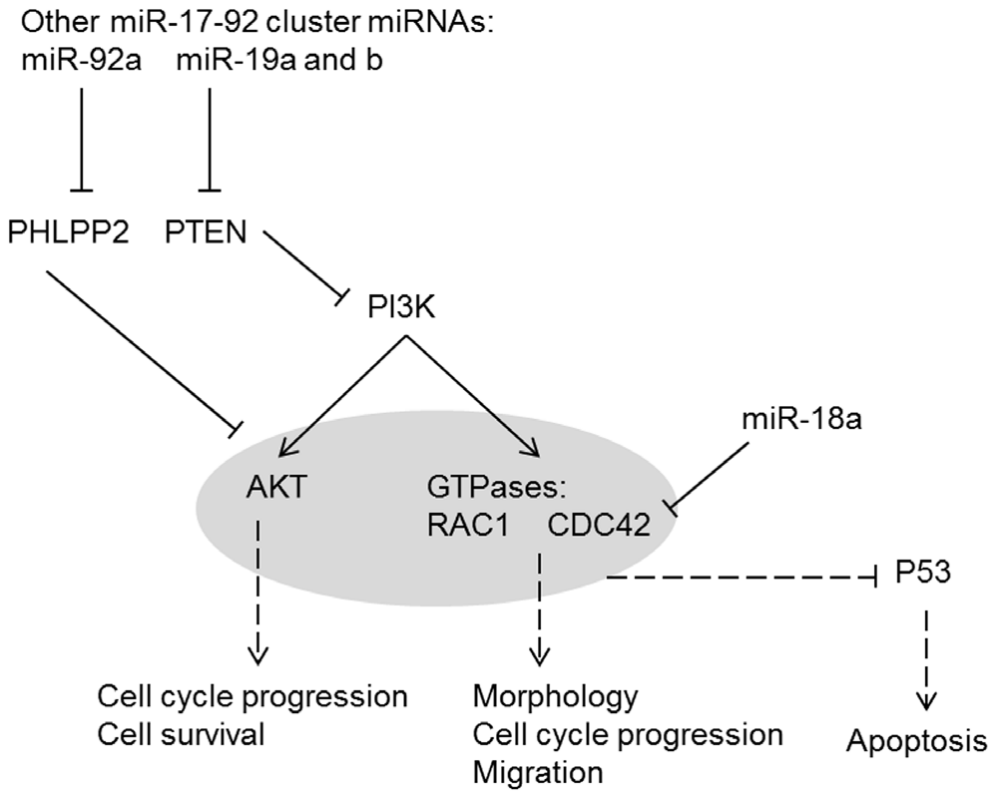
Proposed model of PI3K pathway regulation by miR-18a and other miR-17-92 cluster miRNAs. Some miR-17-92 cluster members activate the PI3K pathway by supressing inhibitors of the pathway [13]; for example, miR-19 directly targets and represses PTEN [12]. The homeostatic function of miR-18a may be achieved through suppression of the PI3K pathway, by targeting *CDC42* and reducing expression.

miR-18a appears to play a homeostatic role in opposing the action of the other miR-17-92 cluster members (Figure 10). The miR-17-92 cluster as a whole has recently been shown to drive cancer development by supressing the expression of inhibitors of the PI3K and NFkB pathways, thereby activating these pathways and promoting cell cycle progression, survival and migration [13]. miR-17-92 cluster members supress the expression of inhibitors of the PI3K pathway, such as PTEN and PHLPP2 [13]. miR-19 in particular has been shown to directly target and repress PTEN, with over-expression of miR-19 significantly down-regulating PTEN mRNA and protein levels [12]. miR-92a has been shown to target PHLPP2 [45], [46]. The activation of the PI3K pathway leads to downstream effects mediated by AKT and Rho GTPases, particularly RAC1 and CDC42; CDC42 is activated by the p85α subunit of PI3K and by AKT [44], [47]. In contrast to the activation of the PI3K pathway by other miR-17-92 cluster miRNAs, miR-18a was shown in this study to inhibit *CDC42* expression, which could reduce activity of this pathway. In addition, P53, an important tumour suppressor, is inhibited by components of the PI3K pathway, including CDC42, RAC1, and the AKT pathway [48], [49]. Knockdown of *CDC42* has been shown to increase and activate P53, leading to apoptosis [48], [49]. Thus, the targeting of *CDC42* by miR-18a may also enhance the pro-apoptotic action of P53.

Inhibition of miR-17-92 downstream pathways, such as the PI3K pathway, has been identified in a rodent model as having therapeutic value in treating cancers with miR-17-92 overexpression [13]. Inhibition or delivery of particular miR-17-92 cluster miRNAs may also have therapeutic potential in human cancers. AntimiR-based therapy could possibly be used to inhibit miR-19a and b and other oncogenic members of the miR-17-92 cluster. This approach has been trialled in humans for treatment of hepatitis C virus infection using a locked nucleic acid antimiR oligonucleotide against miR-122 [50], and has also been used for members of the miR-17-92 cluster in rodents [51].

Alternatively, miRNA replacement therapy could be used to increase levels of miR-18a in colorectal tumours. Human trials of miRNA replacement therapy are commencing, with a miR-34a mimic currently being trialled for the treatment of liver cancer [52]. miRNA replacement therapy would ideally target the tumour of interest, as miRNAs that play protective roles in some tissues may promote disease in others [9]. miR-18a has been shown to have a tumour suppressor role in CRC, in both our study and in another recent study by Fujiya et al (2013) [53]. These two studies present alternative or complementary mechanisms of action. Fujiya et al (2013) postulated that miR-18a acts through a non-canonical mechanism, to induce apoptosis by binding to hnRNPA1 (a protein with possible oncogenic activity) and promoting autophagolysosomal degradation. The study also used an *in vivo* xenograft model to display the inhibitory effects of miR-18a on CRC progression; in this model, tumours in a control group increased in size, whereas in a group with miR-18a administered using viral vectors, tumour growth was almost completely supressed [53]. Fujiya et al (2013) did not investigate the canonical miRNA action of targeting specific mRNAs and supressing translation, and did not identify direct mRNA targets for miR-18a. In contrast, our study showed miR-18a directly targets *CDC42*, and through this inhibition is able to suppress proliferation. miR-18a has also been shown to suppress proliferation in bladder cancer [54], while miR-18b has been implicated as a tumour suppressor in melanoma [55]. In contrast to suggested tumour suppressor roles, an oncogenic role for miR-18a has been suggested in several cancers, including breast and nasopharyngeal cancer [56], [57]. miR-18a has also been shown to target Dicer, a member of the miRNA biogenesis pathway, thereby potentially contributing to altered miRNA expression profiles [57], although it should be noted that the Dicer 3′UTR is littered with binding sites for many miRNAs, which may simply indicate a general feedback loop. miR-18a may have divergent roles in different cancer types, by targeting genes important in specific tissue contexts. In addition to investigating the therapeutic delivery of miR-18a to colorectal tumours, future work could also further investigate why miR-18a expression remains stoichiometrically low compared with other cluster members, how this changes in cancer progression, and whether this processing could be modified. Investigation of the synergistic effect of miR-18a with ML141 may also be warranted, as it demonstrates potential for miRNA therapy to be used in conjunction with other drug treatments, and potential for lower drug concentrations to reduce morbidity associated with chemotherapy.

miR-18a appears to play a tumour suppressor role in CRC cells. In this study, miR-18a reduced proliferation and migration, increased apoptosis, and induced cell cycle arrest in CRC cells. miR-18a had an opposing role to other miR-17-92 cluster members, in particular the key oncogenic miRNAs, miR-19a and b. While other miR-17-92 cluster members activate the PI3K pathway [13], the homeostatic function of miR-18a may be achieved through suppression of this pathway by targeting *CDC42*. Direct inhibition of *CDC42* was identified as a mechanism by which miR-18a reduces cell growth; inhibition of *CCND1* by miR-18a may also assist in this growth-suppression effect. These findings offer new avenues for therapy, although caution is necessary as the action of miR-18a may be tissue specific.
